# Leveraging the unique properties of RNA aptamers to quantify the fraction of receptors occupied by ligands

**DOI:** 10.1016/j.omtn.2026.103016

**Published:** 2026-08-05

**Authors:** George J. Weiner

**Affiliations:** 1 Department of Internal Medicine and Holden Comprehensive Cancer Center, University of Iowa, Iowa City, IA 52242, USA

## Main text

Lord Kelvin said “When you can measure what you are speaking about … you know something about it; but when you cannot measure it … your knowledge is of a meagre and unsatisfactory kind.”^1^ Ligand-receptor interactions are fundamental to biology. They impact cell signaling, immune responses, and the mechanism of action of many therapeutics. Measuring ligands and receptors separately is a cornerstone of biomedical research. Quantifying the fraction of receptors occupied by their ligand has historically been more challenging. Current methods used to quantify receptor saturation by ligand, such as colocalization, fluorescence resonance energy transfer, and proximity ligation assays, are suboptimal. They largely rely on the evaluation of the receptor-ligand proximity,^2^^,^^3^^,^^4^ and not on direct quantification of the interaction. These assays are valuable in some research settings but are semi-quantitative and can be technically challenging to perform, particularly at high-throughput volumes needed for clinical application.^5^^,^^6^^,^^7^

A biomarker platform capable of quantifying the saturation of receptors by their ligands could serve as a powerful research tool in exploring such interactions and has the potential to translate to the clinic and enhance our ability to predict which patients are most likely to respond, or not respond, to a given therapy. We previously reported the development of a novel RNA aptamer-based platform that can be used to quantify the saturation of a receptor by its ligand termed the Ligand-Receptor Complex Aptamer (LIRECAP) assay.^8^^,^^9^ The LIRECAP assay leverages two key aspects of RNA aptamers: (1) the ability of aptamers to fold into structures that can bind to targets in a manner analogous to that of antibodies and (2) the ability of aptamers to be amplified, and quantified, by PCR techniques.

The LIRECAP concept is based on a pair of RNA aptamers with one aptamer that preferentially binds to the ligand-receptor complex and another that binds to unoccupied receptor. Such paired aptamers are of the same length and have the same 5′ and 3′ ends but have distinct central regions that determine their binding specificity and can be detected individually with fluorescent probes. Aptamer pairs selected for the LIRECAP assay should not bind to ligand alone and should not cross-block each other.

**Figure 1:**
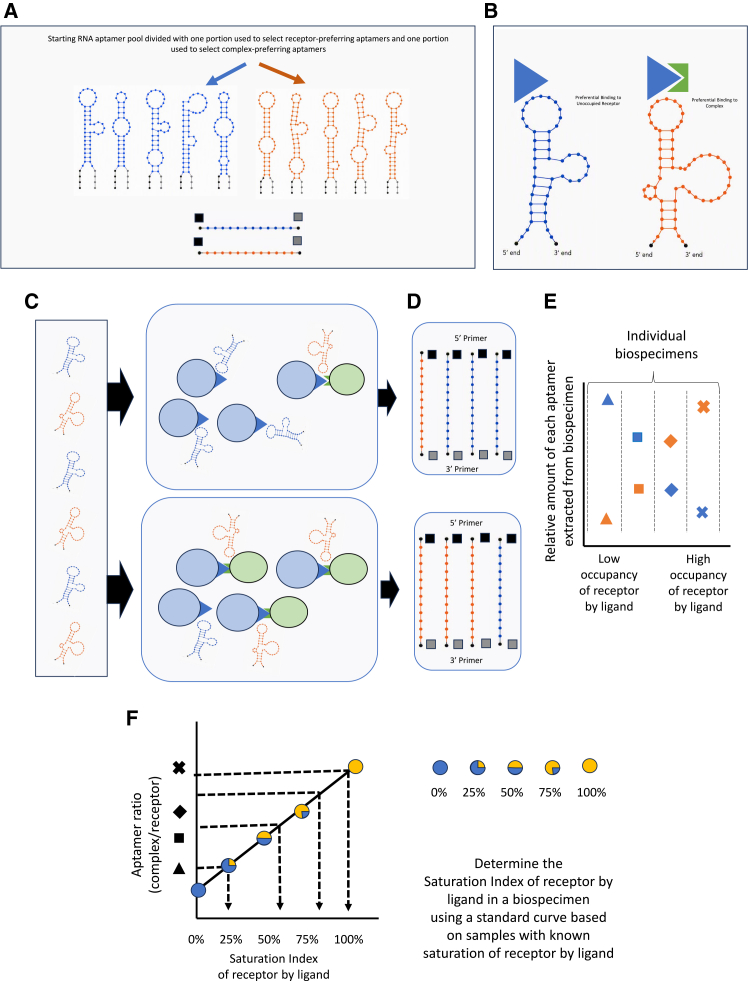
Schematic of the LIRECAP assay (A) Aptamer pools are enriched using a modification of the SELEX technique. Both the unoccupied receptor aptamer pool (blue) and the ligand-receptor complex aptamer pool (orange) come from the same starting library, so selected aptamers are of the same length and have the same 5′ and 3′ ends. (B) Aptamers are selected for the LIRECAP assay including one that preferentially binds to the unoccupied receptor and the other that preferentially binds to the ligand-receptor complex. This specificity can be relative and does not need to be absolute. The two selected aptamers should not bind to the ligand alone and should not cross-block each other. This selection should involve formalin-fixed targets if the goal is to analyze formalin-fixed biospecimens. (C) Equimolar concentrations of the two aptamers are added together to a formalin-fixed biospecimen, samples are washed, and the bound aptamer extracted. (D) Two color RT-qPCRs using a single set of primers is used to quantify the ratio of the two aptamers. (E) Samples with low fractional occupancy of the receptor by ligand will have a low ratio of complex-preferring aptamer to receptor-preferring aptamer, while samples with high fraction occupancy of the receptor by ligand will have a high ratio of complex-preferring aptamer to receptor-preferring aptamer. (F) A standard curve based on samples with known receptor occupancy by ligand is used to convert the ratio of the two aptamers for each biospecimen into the saturation index.

In the LIRECAP assay, the aptamer that preferentially binds to the complex and the aptamer that preferentially binds to the unoccupied receptor are added together to a biospecimen, and the bound aptamer is extracted. Because these two aptamers share 5′ and 3′ ends, they can be expanded concurrently by quantitative reverse-transcription PCR (RT-qPCR) using a single set of primers. Quantifying the ratio of the two aptamers at the same time in the same sample adds a robust level of internal control under conditions that are identical for both aptamers. Through the use of a standard curve composed of samples with known saturation of receptor by ligand, the ratio of the two aptamers as determined by RT-qPCR can be converted to a “saturation index” that reflects the fractional occupancy of the receptor by the ligand in that biospecimen (Figure 1). This approach controls for cross reactivity, since aptamers that preferentially bind to the unoccupied receptor typically have a low level of cross reactivity with the complex and aptamers that preferentially bind to the complex typically have a low level of cross reactivity with the unoccupied receptor. The saturation index is independent of the absolute amounts of receptor or ligand in the sample. Thus, the LIRECAP assay represents a novel, sensitive, and technically straightforward approach to quantifying the saturation of a receptor by its ligand that can be performed in research and clinical laboratories using standard equipment and expertise. If the selection of aptamers is done using formalin-fixed targets, aptamers for the LIRECAP assay can be chosen that are able to react with those targets in formalin-fixed paraffin-embedded tissue, which is standard in clinical pathology.

We came upon this concept when studying soluble CD25 and its ligand IL2.^8^^,^^9^ We recently published in the International Journal of Molecular Sciences, the development of a PD1 LIRECAP assay because of the immense research and potential clinical applicability of an assay that can quantify the saturation of PD1 by PDL1 in formalin-fixed paraffin-embedded tumor tissue.^10^ Anti-PD1 therapy (including antibodies against both PD1 and PDL1) is a mainstay of treatment of multiple cancer types.^11^ It is estimated that approximately 250,000 patients received such therapy in 2024. The US market for anti-PD1/anti-PDL1 antibodies is approximately $28 billion/year. While the overall clinical value of anti-PD1 treatment for many patients is undeniable, current biomarkers used to predict which patients are most likely to benefit from therapy, including tumor mutational burden, PDL1 expression, and T cell infiltration, are of limited value clinically.^12^^,^^13^^,^^14^^,^^15^ Overall, it is estimated that approximately 20%–25% of patients currently treated with anti-PD1 therapy for a variety of malignancies benefit from therapy.^16^ Due to the cost and uncertainty of clinical benefit, many patients who have a real but small chance of responding to anti-PD1 therapy do not receive it. Those who do receive anti-PD1 therapy but do not benefit are exposed to toxicities, delays in use of other potentially effective treatments, and often a devastating financial burden. New and improved approaches to predicting which patients are most likely to benefit, or not benefit, from anti-PD1 therapy are clearly needed. There are data, albeit highly preliminary, based on proximity ligation assays suggesting that saturation of PD1 by PDL1 may indeed be prognostic.^17^

To develop the PD1 LIRECAP assay, we identified one aptamer that preferentially binds to formalin-fixed unoccupied PD1 and another that preferentially binds to the formalin-fixed PD1-PDL1 complex. Formalin-fixed targets were used for two reasons. First, formalin fixation stabilizes the ligand-receptor complex and so prevents the aptamers themselves from influencing the interaction between PD1 and PDL1. Second, as mentioned above, many cancer patients have formalin-fixed paraffin-embedded tumor tissue available from a biopsy or surgery. An assay that can be performed on tissue already available, without requiring an additional clinical procedure, would be of particular clinical value.

Our preliminary studies indicate quantification of PD1 saturation by PDL1 as determined by the PD1 LIRECAP assay correlates closely with PD1-mediated signaling and PD1-PDL1 proximity.^10^ The assay was highly reproducible when individual samples, including formalin-fixed paraffin-embedded clinical biospecimens were evaluated at different times. Clinical biospecimens showed considerable intratumoral heterogeneity when the PD1 LIRECAP assay was performed on distinct slices of tissue obtained from a given clinical biospecimen. This intratumoral heterogeneity was biological and not technical. It is found with other tissue biomarkers and is consistent with the variability in immune infiltrates within various regions of a given tumor. Despite the intratumor heterogeneity in saturation index as determined by the PD1 LIRECAP assay, there were clear differences between tumors with some samples showing a high level of PD1 saturation by PDL1 and others showing less saturation.

With this novel bioassay platform for quantifying PD1 saturation by PDL1 in hand, we are now evaluating whether the saturation index of PD1 by PDL1 in clinical biospecimens, as determined by the PD1 LIRECAP assay, correlates with clinical response to anti-PD1 therapy. We are also developing LIRECAP assays designed to quantify the saturation of a variety of receptors by their ligands that we expect to be useful for research, and potentially clinically as well. A similar approach is being planned to assess target saturation by drugs.

In summary, by leveraging the properties of aptamers in a novel way, we are now able to quantify the saturation of receptors by ligands such as saturation of PD1 by PDL1. To revisit Lord Kelvin again, by enhancing our ability to quantify receptor-ligand interactions, we believe the LIRECAP technology can take our knowledge of receptor saturation by ligand from “meagre and unsatisfactory” to where “we know something about it,” which, in turn, could have significant research and potentially clinical impact.

## Declaration of interests

G.J.W. is an inventor on patent US #10,934,549 and additional submitted patents related to the LIRECAP technology. He is also the founder and has an ownership interest in LIRECAP, Inc., which has financial interest in this technology.

## Declaration of generative AI and AI-assisted technologies in the writing process

Artificial intelligence was used to assist in development of the aptamer illustrations included in Figure 1.
